# Water, sanitation and hygiene (WASH) practices and deworming improve nutritional status and anemia of unmarried adolescent girls in rural Bangladesh

**DOI:** 10.1186/s41043-023-00453-8

**Published:** 2023-11-13

**Authors:** Saira Parveen Jolly, Tridib Roy Chowdhury, Tanbi Tanaya Sarker, Kaosar Afsana

**Affiliations:** 1 BRAC James P Grant School of Public Health, BRAC University, 6th Floor, Medona Tower, 28 Mohakhali Commercial Area, Bir Uttom A K Khandakar Road, Dhaka, 1213 Bangladesh; 2 BRAC Research and Evaluation Division, BRAC, 75 Mohakhali, Dhaka, 1212 Bangladesh

**Keywords:** Adolescent girls, Anemia, Bangladesh, Deworming, Nutritional status, Rural, WASH

## Abstract

**Background:**

In Bangladesh, undernutrition and anemia are more occurrent among adolescent girls. BRAC, the largest non-governmental organization (NGO), has been implementing a community-based nutrition education service package targeting adolescent girls for reducing their undernutrition and anemia.

**Objective:**

We aimed to explore the underlying factors associated with nutritional status and anemia among adolescent girls under the BRAC nutrition program areas to improve their existing intervention package.

**Methodology:**

We conducted a cross-sectional and comparative study in 2016, in 24 *upazilas* of Bogra, Barguna, Comilla, Dinajpur, Feni, Jessore, and Meherpur districts where the BRAC nutrition program was implemented while the remaining 27 *upazilas* of those districts were selected as comparison area. We followed a multistage cluster random sampling for selecting 1620 unmarried adolescent girls aged 10–19 years for interviewing in the intervention and comparison areas. Data were collected on socio-demographic information, dietary intake, morbidity, water, sanitation, and hygiene (WASH) practice, anthropometry, and serum hemoglobin (Hb) level by using a pre-structured questionnaire. The nutritional status of the adolescent girls was expressed as height-for-age Z (HAZ) and body mass index-for-age Z (BMIZ) score, while anemia referred to the serum Hb at the level of below 12 g/dl for adolescent girls. All statistical analyses were done in STATA version 17 (Chicago Inc.).

**Findings:**

The prevalence of stunting (22.9% vs. 22.5%), thinness (12% vs. 14%), and anemia (34.5% vs. 37.3%) exhibited similarities between the intervention and comparison regions. Stunting and thinness were predictors for each other for this population group. Our findings indicated that adolescent girls who were not washing hands with soap after defecation were likely to be stunted [AOR 1.51 (95% CI 1.12–2.04)], and who did not utilize sanitary latrines had an increased likelihood of being thin [AOR 2.38 (95% CI 1.11–5.08)]. Conversely, those who did not watch television [AOR 1.69 (95% CI 1.12–2.56)] and did not have deworming tablets [AOR 1.33 (95% CI 1.07–1.64)] in the 6 months leading up to the interview had a 69% and 33% higher probability of being anemic, respectively.

**Conclusion:**

For sustainable improvement in the undernutrition and anemia of adolescent girls, integration of WASH, consistent administration of deworming tablets and broadcasting awareness programs through television are urgent to scale up the nutrition intervention programs in similar settings like Bangladesh.

## Introduction

Adolescence marks a phase of rapid growth and demands for increased nutritional requirements following early infancy [1, 2]. The World Health Organization (WHO) defines this period as spanning from 10 to 19 years of age [3]. During this time, nutrition-related challenges are aggravated by factors like menstrual blood loss and unforeseen pregnancies [4]. Presently, adolescent fertility contributes to 11% of global births, with 95% of these instances occurring in low- and middle-income countries [4]. Bangladesh, specifically, is home to around 14.4 million adolescent girls, comprising nearly one-fifth of the total population [5]. However, early marriage and teenage pregnancy remain prevalent in Bangladesh, often happening by the age of 16 years, while the average age of first childbirth is 18 years [6]. Hence, it becomes imperative to identify the root causes of undernutrition and anemia among Bangladeshi adolescent girls, to eliminate their adverse impacts on future generations.

Globally, major nutritional problems of adolescent girls are stunting and thinness which occurs due to a lack of optimal food intake [7]. The origins of malnutrition can be traced back to prenatal stages, where evidence highlights the impediment of catch-up growth in low-birthweight (LBW) babies due to inadequate nutrition, resulting in persistent stunting during later adolescence. In Bangladesh, the adolescent phase is marked by a deceleration in the growth rate of height-for-age Z (HAZ) score and body mass index-for-age Z (BMIZ) score [8]. The short stature not only affects pelvic development of adolescent girls but also perpetuates a potential cycle of malnutrition, as stunted adolescent girls could become stunted mothers who may face obstetric complications and give birth to LBW babies [9–13].

Additionally, iron requirement peaks during the age of 14–15 years among adolescent girls due to rapid pubertal growth with a sharp increase in lean body mass, blood volume, and red cell mass [14]. This demand for iron persists even after growth spurts, particularly to compensate for menstrual blood loss during reproductive years [14, 15]. Iron deficiency causes anemia which has some functional consequences, such as cognitive impairment, decreased physical activity, and reduced immunity [16]. Infections and worm infestations serve as primary drivers of impaired nutrient absorption, compromised nutrient transport to target tissues, heightened catabolic losses, and deficiencies in essential micronutrients [17–21]. Therefore, to disrupt this cycle of malnutrition and pave the way for healthier future generations, a promising strategy involves enhancing the nutrition of adolescent girls before conception, requiring strategic investments in their physical and neuro-maturational development [22].

Evidence shows that stunting, underweight, and anemia share common predictors, which can be addressed through various strategic interventions. In the context of Bangladesh, stunting was linked to illiteracy and poverty, while being younger in age, residing in rural areas, experiencing prolonged periods of illness, and facing poverty were associated with underweight among female adolescents who sought treatment in both rural and urban health facilities [23]. In India, inadequate water, sanitation and hygiene (WASH) practices, such as lack of household water facilities, open defecation, and failure to use soap after defecation, were connected to a higher prevalence of stunting and underweight in adolescent girls [24].

Moreover, WASH is an important component for tackling anemia among adolescent girls along with iron‒folic acid supplementation [25], deworming, and co-administration of retinol [26]. In Nepal and Burkina Faso, a 15-month randomized controlled trials targeting nutrition and health components in school children revealed that WASH initiatives and school gardening led to increased consumption of fruits and vegetables, improved hygiene behaviors, and reduced intestinal parasites, consequently contributing to a reduction in anemia [27, 28]. While these studies did not identify effects on stunting and underweight, they highlighted the need for a more comprehensive, nutrition-specific approach to impact anthropometric indices.

In Bangladesh, programs designed for schools may not be sustainable approach for eradicating undernutrition and anemia among adolescent girls due to higher rates of school dropout in rural areas and urban slums, largely attributed to early marriage and societal constraints [29]. The Government of Bangladesh (GoB) is committed to creating a healthy, productive, socially secure, and supportive environment by integrating nutrition education and hygiene promotion into the National Adolescent Health Strategy 2017‒2030, aligning with SDG-2 and SDG-6 [30]. To effectively implement these strategies, collaboration between the GoB and local as well as international non-governmental organizations (i-NGOs) is essential.

Since 1982, BRAC health, nutrition, and population program has prioritized the well-being of under-five children and women. Acknowledging the urgency to enhance nutritional status and to reduce iron deficiency anemia of adolescent girls, BRAC introduced a community-based nutrition education package in 2013. This initiative targets girls in seven rural districts of Bangladesh and involves it’s BRAC community health workers. They delivered key messages on balanced diets, dietary diversity, iron-rich foods, intake of iron tablet, nutritional needs, health, and hygiene practices, including WASH. Additionally, the BRAC WASH program operates in 61 districts across Bangladesh, promoting sanitary practices and handwashing. However, there was a pressing need to assess the undernutrition and iron deficiency anemia among the target groups and understand their requirements. Thus, a baseline survey was conducted to assess the prevalence of undernutrition and anemia among adolescent girls. This study aims to identify key factors associated with undernutrition and anemia, contributing to the refinement of the existing nutrition intervention package.

## Methodology

### Study design, setting, and sampling

**Fig. 1:**
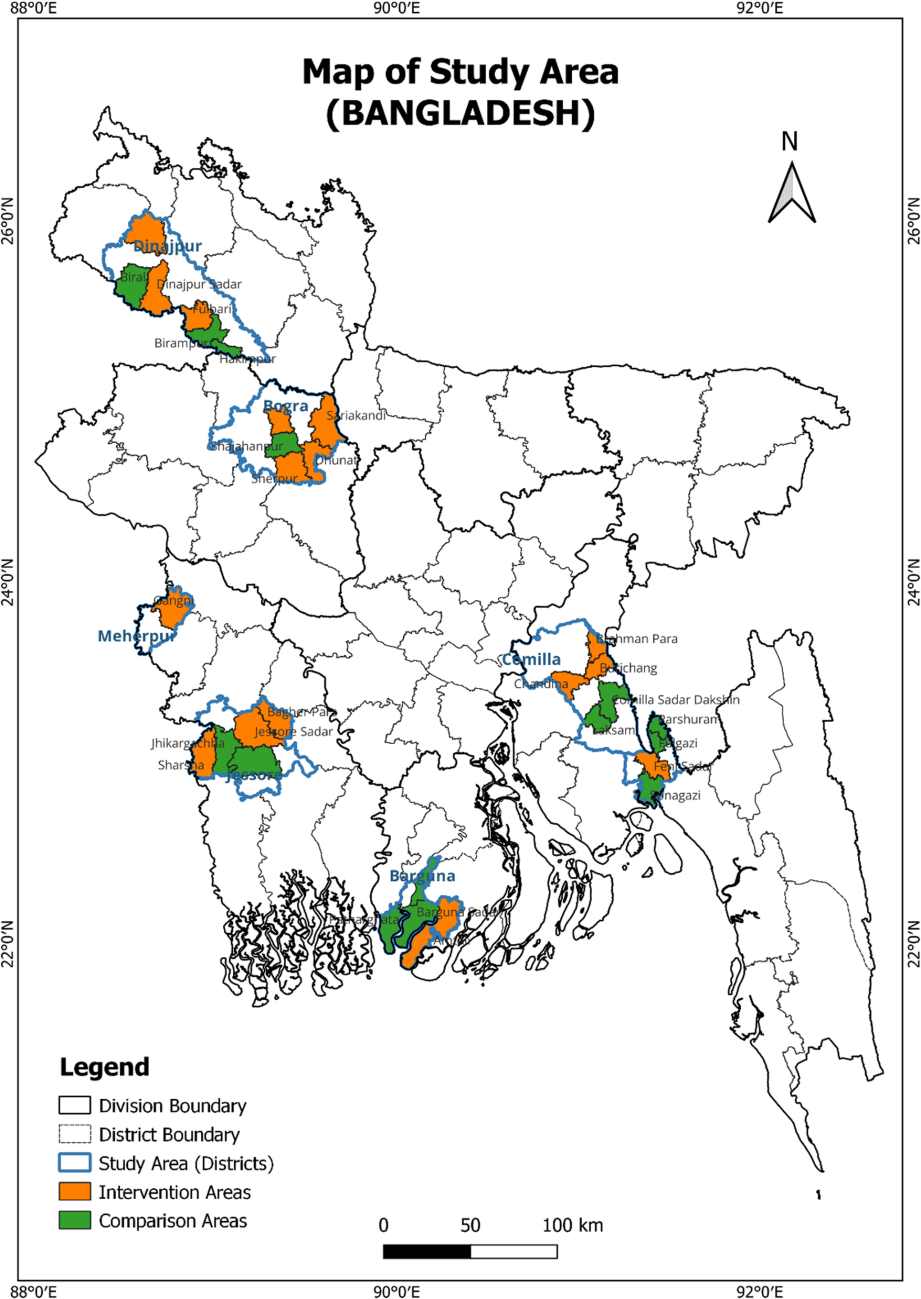
BRAC nutrition program area and comparison area in the map of Bangladesh

**Fig. 2:**
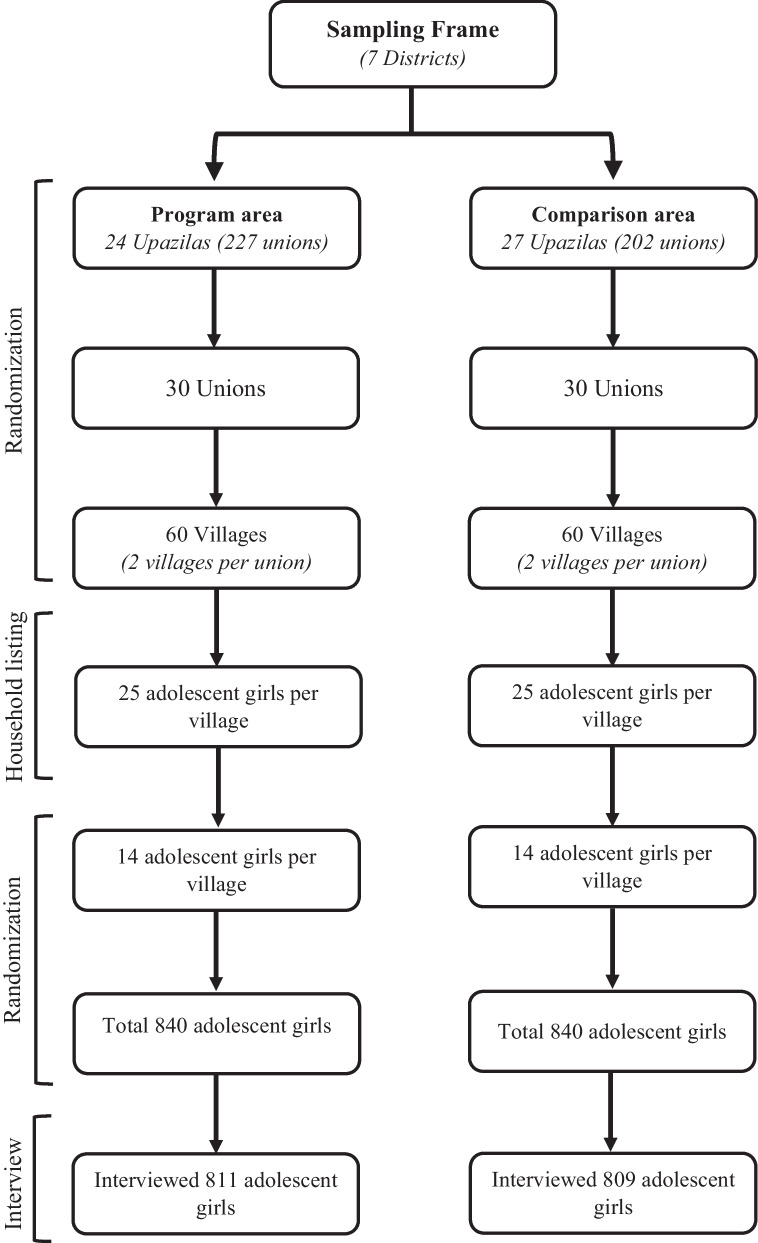
Sampling and randomization for selecting the adolescent girls in intervention and comparison areas

We conducted a cross-sectional and comparative, baseline study, during January-June 2016, in areas where BRAC implemented a nutrition education program to those where it was not implemented. The study was conducted to assess the nutritional status of adolescent girls. The BRAC nutrition education intervention was implemented in 24 *upazilas* of Bogra, Barguna, Comilla, Dinajpur, Feni, Jessore and Meherpur districts (Fig. 1). These *upazilas* were selected as intervention areas. The remaining non-intervention 27 *upazilas* of the seven districts were selected as comparison areas (Fig. 2). From 24 intervention *upazilas* (227 unions), 30 unions, and from each union, two villages were selected randomly. In the comparison areas, 27 *upazilas* (202 unions), 30 unions, and 60 villages were selected randomly by following the same procedure. In each village, a house-to-house census was conducted to enlist 25 unmarried adolescent girls aged 10–19 years. Later out of 25 adolescent girls, 14 were selected randomly for an interview. Finally, a total of 811 and 809 adolescent girls were interviewed from intervention and comparison areas, respectively.

### Sample size calculation

Based on the prevalence of anemia in adolescent girls (51%; [31]) in Bangladesh, this study was interested in detecting the prevalence of undernutrition and anemia with a 95% confidence level and 5% precision. The sample size was calculated by using the formula, $n = \frac{{Z\alpha^{2} \times p \left( {1 - p} \right)}}{{m^{2} }}$ [32–35]. The calculated sample size was multiplied by two to adjust the design effect. Finally, the total number of adolescent girls in each study area were determined at 799.

### Data collection tools and quality control

A pre-structured questionnaire was used for this survey. The validated questionnaires of the NNP baseline survey 2004 [36] and the national micronutrients survey [37] were considered as aids during the development of the survey tool. The content of the questionnaire was reviewed and modified in alignment with the study objectives. Twenty-eight female interviewers and seven male field supervisors were recruited based on their prior experience in the nutrition survey for in-person interviews. They received comprehensive training for 9 days, followed by field practices for 2 days along with reporting feedback for 2 days to adhere to the questionnaire and its format strictly, with the same degree of questioning on the objective measurement for both the intervention and comparison areas.

### Dietary diversity score

The Dietary diversity of adolescent girls was calculated by using the method of minimum dietary diversity for women (MDD-W) [38]. The 10 food groups, namely starchy foods; pulse; nuts and seeds; dairy; fish, meat & poultry; green leafy vegetables; yellow fruits and vegetables; other fruits and other vegetables were first summed into a score ranging from 0 to 10. Later, the DDS was segregated into three categories; namely low, average and high which refer to intake of one-three, four-six and ≥ seven food groups , respectively.

### Food consumption data collection

A validated semi-quantitative Food Frequency Questionnaire (FFQ) was used to obtain a 7-day food consumption pattern [37]. The study focused on the consumption of iron-rich foods by adolescent girls. Our study employed a range of tools to quantify shared plate eating habits though the tools for assessing shared plate eating. We also made use of various commonly used plates, bowls, spoons and cups, along with varying proportions of dry bean’s packets to help participants better understand serving sizes. In the initial stages, we created bean packets matching the portion sizes of different foods, such as rice, vegetables, and dhal. Participants were shown these packets alongside their locally familiar plates, allowing them to indicate their typical rice intake proportions using the packets of beans as reference. This approach enabled us to gauge their eating patterns accurately. Subsequently, we assessed their dietary intake based on their responses. Additionally, we employed a unique method involving round pieces of plain brown hard packaging paper to visually represent the size of hand-made roti. By observing participants' interaction with the paper, we could estimate their flour intake accurately. Collectively, our methodology was tailored to the local context and facilitated a comprehensive understanding of shared plate eating habits. Through these innovative techniques, we gained insights that traditional survey methods might not have captured fully. While no methodology is without limitations, our approach proved effective in assessing dietary intake and shared plate eating behaviors. Raw food weight was calculated by using appropriate conversion factors [39]. Nutrient values were calculated per 100 g of raw food consumed using the food composition table on Bangladeshi food [40].

### Anthropometric measurement

Body weight was measured to the nearest 100 g using a portable electronic weighing scale (TANITA Corporation Japan, model HD—318). The girls were weighed barefoot. Height was measured from barefoot to the nearest 0.1 cm using a locally made height board. MUAC of the adolescent girls was measured nearest to the 0.2 mm using MUAC tape produced by Teaching Aides at Low Cost (TALC). Anthropometric data were converted to Z-scores by using the WHO growth standard (Anthro, 2005). Adolescent girls’ nutritional status was explained by HAZ and BMIZ [41].

### Hemoglobin measurement

Two field research assistants were assigned the task of measuring serum hemoglobin (Hb) levels. One assistant collected the blood specimens, while the other recorded the readings. The measurement of Hb was conducted using portable Hemocue β‒Hemoglobin Photometers. Capillary blood samples were obtained from the middle finger through a finger-prick using a lancet. The initial two blood drops were discarded using a dry gauze to induce a natural blood flow. The third drop of blood was carefully collected onto a microcuvette, which was then placed into the photometer. The reading of Hb was recorded once the displayed measurement stabilized. The Hemocue β‒Hemoglobin microcuvettes served as both reagent vessels and measuring devices. The puncture site was cleaned using a cotton bud soaked in hexisol antiseptic. Following this, the finger was wrapped with an adhesive bandage. To ensure safety, the field research assistants consistently wore latex gloves throughout the procedure, changing gloves for each respondent’s Hb measurement. After usage, the lancet, microcuvette, gloves, gauze, and cotton were disposed of following standard biohazard protocols. Anemia of the unmarried adolescent girls was defined as hemoglobin level below 12 mg/dl [42].

### Statistical analysis

A wealth index based on the ownership of household assets is widely recognized as a proxy for gauging household economic status [43, 44]. To measure the wealth index, some variables such as property, household assets, household construction materials, water, sanitation, and fuel supply were converted to dichotomous variables. Later, factor analysis was used to assign weighting values to indicator variables. A wealth quintile was constructed using the ranking procedure. Analysis of the parametric continuous variables was performed using the student’s *t*-test and result showed as mean ± standard deviation (SD) with a *p* value. All the categorical variables were analyzed using the Chi-square (χ^2^) test, and the result was expressed as a percentage, a number, and a *p* value. The association between indicators and predictors was analyzed using the multivariate logistic regression model, and data were expressed as an adjusted odds ratio (AOR) with a 95% confidence interval (CI). Initially, all the continuous variables like HAZ, BMIZ, and Hb levels were converted to dichotomous variables (0,1) according to their cutoffs. Later intake of energy, protein, iron was converted to dichotomous based on their median value. Other predictor variables were also converted to “0” and “1”. Since we found that there was no significant difference in the characteristics and in the outputs of logistic regression analysis between intervention and comparison areas, we performed the multivariate logistic regression by compiling both areas to improve the precision of the estimates. The Pearson Chi-square test was employed to determine if there was any connection between the two categorical variables. However, it did not quantify the strength or extent of this association. In contrast, the Goodman–Kruskal gamma test is akin to a correlation coefficient in that it assesses the level of association between two categorical variables, primarily ordinal ones. It generates values ranging from − 1 to 1, where − 1 signifies a robust negative association or complete opposition, 1 suggests a strong positive association or perfect agreement, and 0 denotes no association, much like the interpretation of the Pearson correlation coefficient. When we were dealing with two nominal variables, Creamer’s V was utilized to gauge the correlation coefficient. This coefficient spans from 0 to 1, where 0 signifies no association and 1 indicates a strong association. Conversely, for ordinal variables, the Goodman–Kruskal Gamma coefficient was employed to gauge the degree of association. This coefficient also varies from − 1 to + 1. Here, − 1 denoted a strong negative association or complete reversal, + 1 signified a strong positive association or perfect agreement, and 0 indicated no association. We also found some predictors such as age, education, and wealth quintiles from earlier studies. We assumed that young age, illiteracy, low socioeconomic status, no access to WASH practices and deworming tablets, low consumption of nutrients, undernutrition, not exposed to mass media and anemia were associated with stunting, thinness and anemia of the adolescent girls. We conducted an assessment of multicollinearity among the independent variables in our models, specifically for HAZ, BMIZ, and the model involving anemia. The variance inflation factor (VIF) values for these variables ranged from 1.01 to 2.91 for HAZ, 1.01 to 2.92 for BMIZ, and 1.01 to 2.90 for the anemia model. It is important to note that most of the literature suggests that VIF values exceeding “5” might indicate a potential collinearity issue [45–47]. However, our analysis reveals that the independent variables utilized in all nine distinct models do not exhibit significant collinearity concerns. The fact that the Prob > LR values for all three models are statistically significant (*p* < 0.05) indicating that these models are well fitted. This analysis was performed using STATA version 17 (Chicago Inc.). Significance was taken at *p* < 0.05.

## Results

### Socio-demographic status, WASH practice and morbidity

Table 1 illustrates the socioeconomic condition, WASH practice, and morbidity of adolescent girls in both intervention and comparison areas. We observed that their average age was 13 years and almost all of them could read and write in both areas. Additionally, they were equally distributed in different strata of wealth quintiles. Their average weight and height were 36 kg and 146 cm, respectively. In the intervention area, 97% and in comparison, 96% of adolescent girls were using the sanitary latrine. We observed differential handwashing practices among adolescent girls at different critical points. About 76% of adolescent girls washed both hands with soap after defecation, but this practice was below 30% before taking food in both areas. Almost all adolescent girls in both areas wore sandals before going to the latrine. We found that only one out of ten adolescent girls in both areas used hygienic sanitary napkins during menstruation. Coverage of consumption of deworming tablets was 51.9% and 55.6% in the intervention and comparison areas, respectively. Prevalence of common diseases such as fever (17.8% vs. 25.2%; *p* < 0.0001), and cough/cold (25.6% vs. 32.1%; *p* < 0.0001), during 2 weeks before the interview, was lower among girls in the intervention while the prevalence of diarrhea, dysentery, and skin diseases among them was comparable between the study areas (Table 1).

**Table 1:** Socio-demographic condition, hygiene practice and health of the adolescent girls by study areas

Variables	Study area		*p* value
	Intervention n = 811	Comparison n = 809	
*Socio-demographic characteristics*			
Age in year, mean ± SD**	13.41 ± 2.13	13.43 ± 2.24	0.889
Household size, mean ± SD**	5.18 ± 1.69	4.86 ± 1.47	0.000
Can read and write, % (n)*	97.7 (792)	98.0 (793)	0.613
Educational status, % (n)*			
None	0.7 (6)	0.4 (3)	0.021
Primary incomplete	26.6 (216)	23.4 (23.4)	
Primary complete	17.3 (140)	14.1 (114)	
Secondary incomplete	47.8 (388)	51.2 (414)	
Secondary or more	7.5 (61)	11.0 (89)	
Primary occupation, % (n)*			
Student	91.5 (742)	92.5 (748)	0.204
Others^a^	8.5 (69)	7.5 (61)	
Wealth index, % (n)*			
Poorest	19.4 (157)	20.6 (167)	0.339
Poor	18.9 (153)	21.1 (171)	
Middle	19.5 (158)	20.4 (165)	
Rich	20.3 (165)	19.7 (159)	
Richest	21.9 (178)	18.2 (147)	
*Water, sanitary and hygiene (WASH) practices*			
Used sanitary latrine, % (n)*	97.0 (787)	96.2 (779)	0.401
Washed both hands with soap before having food, % (n)*	28.1 (228)	26.7 (216)	0.339
Washed both hands with soap after defecation, % (n)*	76.0 (616)	78.9 (638)	0.099
Wore sandal before going to toilet, % (n)*	96.4 (782)	96.5 (781)	0.900
Number of girls whose menstruation had started, N	558	543	
Used sanitary napkin during menstruation, % (n)*	10.2 (57)	9.9 (54)	0.882
*Deworming and morbidity*			
Received deworming tablet within last 6 months, % (n)*	51.9 (410)	55.6 (438)	0.340
Prevalence of common morbidities during last 2 weeks of interview, % (n)*			
Fever	17.8 (144)	25.2 (204)	0.000
Cold/cough	25.6 (208)	32.1 (260)	0.004
Diarrhea	1.1 (9)	1.2 (10)	0.813
Dysentery	0.5 (4)	0.7 (6)	0.523
Skin disease	1.2 (10)	1.6 (13)	0.525

*Chi-square test

**Student *t-*test

^a^Response was taken only from the girls whose menstruation already had started

### Dietary diversity and nutrient intake

Our findings revealed that adolescent girls in both areas had at least four food groups out of ten during the last 24 h of the interview. About 60% of adolescent girls in the intervention area and 59% in the comparison area had 4–6 food groups, during the last 24 h of the interview. Intake of green leafy vegetables by adolescent girls was higher in the comparison area compared to the intervention area (20.5% vs. 30.3%; *p* < 0.0001). A negligible number of adolescent girls in both areas had organ meat during the last 24 h preceding the interview; three-fourths of them had either fish, meat, or poultry. We observed that in both areas these girls had similar amounts of macro- and micronutrients. Importantly, vitamin A and vitamin C consumption were higher in the comparison area compared to the intervention area (Table 2). Doses of intake of iron supplementation were similar in both areas during the last 6 months (Table 2).

**Table 2:** Dietary diversity and nutrients intake and of the adolescent girls by study areas

Variables	Study area		*p* value
	Intervention n = 811	Comparison n = 809	
*Dietary diversity*			
Average number of food groups intake during last 24 h, mean ± SD**	3.91 ± 1.25	3.97 ± 1.24	0.889
Dietary diversity score during last 24 h, n (%)*			
1–3 food groups (low)	37.85 (307)	38.44 (311)	0.431
4–6 food groups (average)	59.93 (486)	58.54 (472)	
7–10 food groups (high)	2.22 (18)	3.21 (26)	
*Had vitamin and iron rich foods during last 24 h*			
Vitamin A rich dark green leafy vegetable, n (%)*	20.5 (166)	30.3 (245)	0.000
Organ meat, n (%)*	1.5 (12)	1.2 (10)	0.672
Fish, meat, poultry, n (%)*	73.7 (598)	74.8 (605)	0.630
*Average nutrient intake during last 1 week*			
Energy, in kcal/day, mean (95% CI)**	1344.29 (1357.64–1418.94)	1403.61 (1375.78–1431.43)	0.468
Protein, in g/day, mean (95% CI)**	45.61 (44.37–46.85)	46.36 (45.26–47.46)	0.375
Fat, in g/day, mean (95% CI)**	14.28 (13.78–14.78)	14.28 (13.77–14.29)	0.998
Carbohydrate, in g/day, mean (95% CI)**	260.37 (254.64–266.11)	264.34 (258.83–269.84)	0.328
Calcium, in mg/day, mean (95% CI)**	635.38 (550.01–720.75)	745.07 (667.48–822.30)	0.062
Iron, in g/day, mean (95% CI) **	7.78 (7.5–8.07)	8.65 (8.22–9.07)	0.001
Zinc, in mg/day, mean (95% CI)**	8.85 (8.31–8.78)	8.68 (8.44–9.07)	0.445
Vitamin A, in µg/day, mean (95% CI)**	167.95 (151.88–184.02)	233.01 (207.14–258.19)	0.000
Thiamin, in mg/day, mean (95% CI) **	1.19 (1.16–1.22)	1.18 (1.15–1.21)	0.801
Riboflavin, in mg/day, mean (95% CI)**	0.64 (0.61–0.67)	0.67 (0.62–0.71)	0.365
Vitamin C, in mg/day, mean (95% CI)**	65.00 (61.06–68.93)	71.09 (67.04–75.14)	0.034
*Intake iron supplementation*			
Intake of iron supplement during last 1 month, %(n)*	5.2 (42)	4.4 (36)	0.493
Frequency of taking iron supplement, %(n)*			
Daily	23.8 (10)	38.9 (14)	0.322
7 days	33.3 (14)	30.6 (11)	
< 7 days	42.9 (18)	30.6 (11)	

*Chi-square test

**Student *t-*test

Hrs = Hours

### Nutritional status

**Fig. 3:**
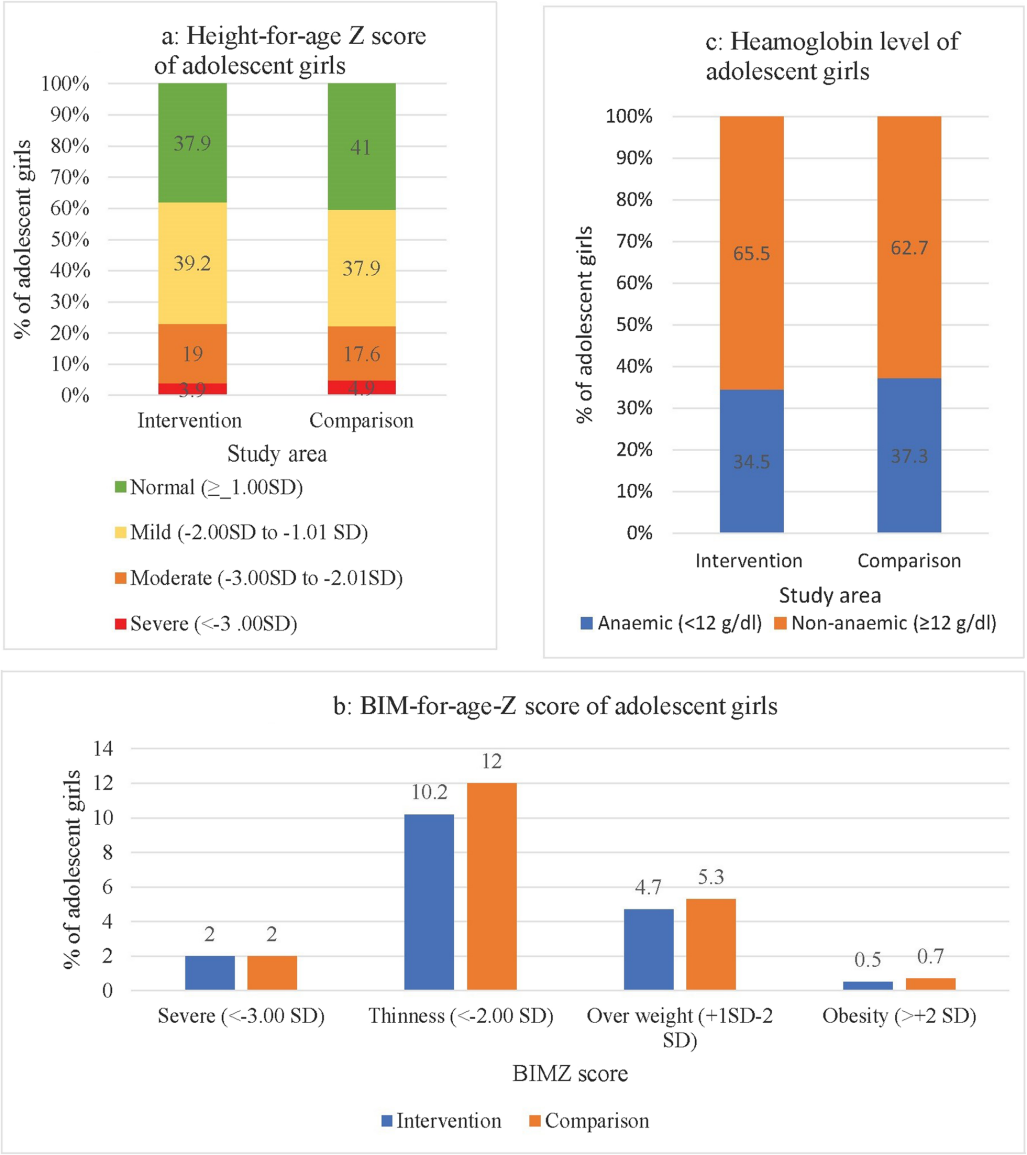
Comparison of nutritional status of the adolescent girls by study areas

The average weight, height, MAC, BMI, HAZ, BMIZ, and Hb levels of adolescent girls were similar in both study areas (Table 3). Prevalence of stunting (22.9% vs. 22.5%; *p* > 0.05) and thinness (10.2% vs. 14%; *p* > 0.05) were comparable between study areas (Fig. 3a, b). The prevalence of anemia was slightly higher in the comparison area compared to the intervention area (34% vs. 37%; Fig. 3c).

**Table 3:** Average nutritional status of adolescent girls by study area

Variables	Study area		*p* value**
	Intervention n = 811	Comparison n = 809	
	Mean±SD	Mean±SD	
Weight in kg	38.36 ± 8.29	38.50 ± 8.85	0.742
Height in cm	146.57 ± 8.26	146.56 ± 8.46	0.985
MACin mm^a^	213.56 ± 28.49	213.42 ± 29.72	0.923
BMIin kg/m^2b^	17.70 ± 2.76	17.74 ± 2.98	0.793
HAZ-score^c^	− 1.27 ± 1.07	1.24 ± 1.05	0.516
BMIZ^d^	− 0.71 ± 1.07	− 0.72 ± 1.11	0.982
Hb in g/dl	12.4 ± 1.3	12.3 ± 1.3	0.529

**Student* t* test

^a^Mid arm circumferences

^b^*BMI* Body mass index

^c^Height-for-age Z score

^d^BMI-for-age Z score

### Predictors of stunting, thinness and anemia

The findings of the multivariate logistic regressions revealed that predictors of stunting, thinness, and anemia exhibited variations between intervention and comparison areas (Table 4). For instance, adolescent girls aged < 13 years had a reduced likelihood of experiencing stunting [AOR 0.43 (95% CI 0.32–0.57)]. This pattern was consistent in both the intervention [AOR 0.39 (95% CI 0.26–0.59)] and comparison [AOR 0.46 (5% CI 0.31–0.68)] areas (Table 4). In contrast, girls who lacked reading and writing skills exhibited a higher prevalence of stunting compared to literate girls [AOR 2.82 (95% CI 1.39–5.70)]. A similar association was observed in the comparison area [AOR 4.39 (95% CI 1.51–12.70)], though not in the intervention area. Similarly, adolescent girls who did not use soap for handwashing after defecation had a 51% increased likelihood of experiencing stunting [AOR 1.51 (95% CI 1.12–2.04)], which was higher for the girls in the intervention area [AOR 2.03 (1.33–3.08)]. Within the intervention area, adolescent girls with iron intake below 7 mg/day had a 56% higher chance of stunting compared to those consuming iron at or above 7 mg/day [AOR 1.56 (95% CI 1.008–2.43)]. Notably, a low BMIZ score emerged as a strong predictor of stunting among all adolescent girls [AOR 1.76 (95% CI 1.44–2.53)], as well as for girls in the comparison area [AOR 2.36 (95% CI 1.44–3.85)] (Table 4).

**Table 4:** Association of stunting, thinness and anemia with predictor variables (multivariate logistic regression)

Predictors	Stunting HAZ < − 2.00 SD = 1; HAZ ≥ − 2.00SD = 0)			Thinness (BMIZ < 2.00SD = 1; BMIZ ≥ − 2.00 SD = 0)			Anemia (Hb < 12 g/dl = 1; Hb ≥ 12 g/dl = 0		
	All model 1	Intervention model 1a	Comparison model 1b	All model 2	Intervention model 2a	Comparison model 2b	All model 3	Intervention model 3a	Comparison model 3b
	AOR (95% CI)	AOR (95% CI)	AOR (95% CI)	AOR (95% CI)	AOR (95% CI)	AOR (95% CI)	AOR (95% CI)	AOR (95% CI)	AOR (95% CI)
Age, in years (< 13 yrs = 1, ≥ 13 yrs = 0)	0.43 (0.32–0.57)*	0.39 (0.26–0.59)*	0.46 (0.31–0.68)*	**2.41 (1.72–2.66)**	**1.97 (1.19–3.26)**	**2.83 (1.77–4.53)**	**1.26 (1.01–1.57)**	1.25 (0.89–1.74)	1.31 (0.96–1.78)
Can read and write (Can = 0, cannot = 1)	2.82 (1.39–5.70)*	1.87 (0.69–5.02)	4.39 (1.51–12.70)*	0.77 (0.22–2.66)	1.30 (0.27–6.26)	0.33 (0.40–0.73)*	0.78 (0.37–1.62)	0.47 (1.53–1.48)	1.00 (0.55–4.62)
Socioeconomic status (1st quintile = 0)									
2nd Quintile	1.05 (0.71–1.56)	1.58 (0.89–2.81)	0.81 (0.46–1.42)	1.38 (0.79–2.41)	1.60 (0.69–3.73)	0.90 (0.44–1.86)	1.15 (0.82–1.62)	0.69 (0.41–1.50)	1.87 (1.16–3.00)*
3rd Quintile	1.45 (0.97–2.18)	1.78 (0.97–3.26)	1.43 (0.82–2.49)	1.70 (0.93–3.11)	2.03 (0.87–4.73)	1.06 (0.50–2.26)	1.35 (0.91–2.01)	1.13 (0.67–1.91)	1.66 (1.006–2.70)*
4th Quintile	0.80 (0.50–1.29)	1.09 (0.55–2.18)	0.66 (0.34–1.27)	1.02 (0.66–1.57)	2.02 (0.78–4.19)	1.66 (0.74–3.73)	1.35 (0.91–2.01)	1.07 (0.60–1.90)	1.71 (0.97–3.01)
5th quintile	0.93 (0.56–1.56)	1.15 (0.53–2.46)	0.95 (0.47–1.93)	1.01 (0.72–1.42)	1.42 (0.48–4.19)	0.87 (0.33–2.24)	1.81 (1.17–2.80)*	1.52 (0.80–2.87)	2.30 (1.24–4.24)*
Watched television (Watched = 0, did not = 1)	0.91 (0.66–1.27)	1.03 (0.54–1.66)	1.16 (0.73–1.84)	1.02 (0.66–1.57)	1.08 (0.57–2.04)	0.97 (0.54–1.75)	**1.54 (1.16–2.05)**	1.69 (1.12–2.56)*	1.41 (0.95–2.11)
Drunk safe water (drink = 0, did not = 0)	1.02 (0.79–1.32)	1.11 (0.76–1.63)	0.84 (0.59–1.20)	1.01 (0.66–1.57)	1.09 (0.65–1.84)	1.002 (0.63–1.75)	1.13 (0.90–1.40)	1.12 (0.81–1.56)	1.17 (0.86–1.58)
Used sanitary latrine (used Sanitary latrine = 0; did not = 1)	0.73 (0.38–1.40)	1.48 (0.55–4.00)	1.24 (0.52–2.99)	**2.38 (1.11–5.08)**	3.45 (1.07–11.07)	1.79 (0.63–5.05)	0.74 (0.39–1.40)	0.30 (0.09–0.95)*	1.22 (0.86–1.58)
Used soap after defecation (used soap = 0; did not = 1)	1.51 (1.12–2.04)*	2.03 (1.33–3.08)*	1.15 (0.74–1.78)	0.90 (0.60–1.38)	1.01 (0.56–1.83)	0.90 (0.51–1.59)	0.96 (0.74–1.26)	0.85 (0.57–1.26)	1.22 (0.54–2.77)
Used soap before eating (used soap = 0; did not = 1)	0.89 (0.66–1.19)	1.19 (0.79–1.79)	1.17 (0.76–1.80)	1.38 (0.92–2.05)	1.50 (0.84–2.67)	1.28 (0.73–2.25)	1.18 (0.92–1.26)	1.08 (0.77–1.53)	1.12 (0.76–1.64)
Wore sandal before going to toilet (wore sandal = 0; did not = 1)	0.94 (0.49–1.78)	0.80 (0.30–2.11)	1.36 (0.56–3.32)	1.54 (0.74–3.20)	1.61 (0.57–4.56)	1.68 (0.58–4.86)	1.14 (0.66–1.92)	1.84 (0.85–3.96)	1.29 (0.90–1.85)
Received deworming tablet (received = 0; did not = 1)	1.07 (0.83–1.37)	0.76 (0.52–1.09)	1.09 (0.76–1.55)	0.88 (0.63–1.24)	0.64 (0.39–1.07)	1.14 (0.71–1.81)	1.33 (1.07–1.64)*	1.36 (0.99–1.86)	0.53 (0.22–1.27)
Protein intake (< 43.42 g = 1, ≥ 43.42 g = 0)	1.14 (0.81–1.61)	1.41 (0.90–2.21)	0.73 (0.48–1.09)	1.21 (0.77–1.90)	1.06 (0.51–2.19)	1.34 (0.75–2.41)	0.90 (0.67–1.20)	1.09 (0.68–1.73)	0.53 (0.22–1.27)
Iron intake (< 7.03 g = 1; ≥ 7.03 g = 0)	1.33 (0.99–1.79)	1.56 (1.008–2.43)*	1.27 (0.85–1.89)	0.94 (0.63–1.39)	1.24 (0.67–2.28)	0.77 (0.45–1.31)	1.15 (0.89–1.48)	1.47 (1.002–2.16)*	1.33 (0.18–1.81)
Energy intake (< 1351 kcal = 1, ≥ 1351 = 0)	0.76 (0.55–1.05)	1.34 (0.84–2.16)	1.29 (0.81–2.03)	1.34 (0.88–2.05)	1.34 (0.71–2.53)	1.46 (0.81–2.62)	0.91 (0.69–1.20)	0.83 (0.55–1.25)	0.70 (0.48–1.04)
Received iron tablet (Yes = 1, Did not = 0)	–	–	–	–	–	–		1.49 (0.72–3.09)	2.09 (0.92–4.73)
Stunting (HAZ < − 2.00 SD = 1, HAZ ≥ − 2.00 SD = 0)	–	–	–	1.73 (1.20–2.50)*	1.20 (0.69–2.09)	2.32 (1.41–3.81)*	1.10 (0.86–1.42)	1.04 (0.72–1.40)	1.14 (0.80–1.63)
Anemia (Hb < 12 g/dl = 0,Hb ≥ 12 g/dl = 0)	1.10 (0.86–1.42)	1.04 (0.72–1.50)	1.14 (0.80–1.62)	1.23 (0.88–1.71)	1.12 (0.68–1.84)	1.34 (0.85–2.10)	–	–	
Thinness (BMIZ < 2SD = 1, BMIZ ≥ 2SD = 0)	1.76 (1.22–2.53)*	1.25 (0.73–2.19)	2.36 (1.44–3.85)*	–	–	–	–		

The use of "bold" in this context signifies that when p-value is less than 0.05, it indicates that the null hypothesis is not contained within the 95% confidence interval (CI)

Model 1: Deviance (1601)—1647.918; LR (18)—88.147; Prob > LR—0.000; Variance of error-3.290; AIC: 1.041; AIC*n—1685.918; BIC—10,183.762 BIC′—44.877

Model 1a: Deviance (1601)—812.201; LR (18)—61.222; Prob > LR—0.000; Variance of error-3.290; AIC: 1.048; AIC*n—80.201; BIC—4492.827; BIC′—59.346

Model 1b: Deviance (1601)—811.891; LR (18)—50.706; Prob > LR—0.000; Variance of error-3.290; AIC: 1.051; AIC*n—849.891; BIC—4477.790; BIC′—69.818

Model2: Deviance (1601)—1068.583; LR (18)—61.633; Prob > LR—0.000; Variance of error-3.29; AIC: 0.683; AIC*n—1106.583; BIC—10,763.097; BIC′—71.391

Model 2a: Deviance (1601)—506.838; LR (18)—28.747; Prob > LR—0.052; Variance of error-3.290; AIC: 0.052; AIC*n—544.838; BIC—4798.190; BIC′—91.822

Model 2b: Deviance (1601)—550.524; LR (18)—42.842; Prob > LR—0.001; Variance of error-3.290; AIC: 0.727; AIC*n—588.524; BIC—4739.157; BIC′—77.683

Model3: Deviance (1601)—2083.622; LR (18)—32.068; Prob > LR—0.022; Variance of error-3.381; AIC: 1.310; AIC*n—2121.622; BIC—9748.058; BIC′—100.955

Model 3a: Deviance (1601)—2083.622; LR (18)—32.068; Prob > LR—0.022; Variance of error-3.290; AIC: 1.310; AIC*n—2121.622; BIC—9748.058; BIC′—100.955

Model 3b: Deviance (1601)—1015.059; LR (18)—30.253; Prob > LR—0.035; Variance of error-3.290; AIC: 1.298; AIC*n—1053.059; BIC—4289.970; BIC′—90.316

AOR, Adjusted odds ratio; ‘0’, Reference; –, Was not in the model

*p < 0.05

Similarly, distinct patterns of thinness were evident between the intervention and comparison areas (Table 4). Thinness was more likely to be prevalent among younger (< 13 years) adolescent girls across all regions [AOR 2.41 (95% CI1.72–2.66)] as well as in intervention [AOR 1.97 (95% CI 1.19–3.26) and comparison [AOR 2.83 (95% CI 1.77–4.53)] areas. Surprisingly, illiteracy did not appear to be correlated with thinness in the comparison area [AOR 0.33 (95% CI 0.40–0.73)]. While the lack of access to a sanitary latrine was associated with thinness among adolescent girls [AOR 2.38 (95% CI 1.11–5.08)]. Interestingly, a connection was found between stunting and thinness in the comparison area [AOR 2.32 (95% CI 1.41–3.81)] and across the areas [AOR 1.73 (95% CI 1.20–2.50)].

Anemia was likely to be associated with younger age of the adolescent girls across the areas [AOR 1.26 (96% CI 1.01–1.57)] (Table 4). We observed an increasing trend of anemia in the different segments of the wealth quintile of adolescent girls in the comparison area. Notably, adolescent girls in the 5th quintile were more likely to be anemic compared to those in other wealth quintile segments [AOR 1.54 (95% CI 1.16–2.05)]. In both the intervention and all other areas, girls who lacked exposure to television had a higher likelihood of being anemic [AOR 1.69 (95% CI 1.12–2.56)], as did those with a low level of iron intake (< 7 mg/day) in the intervention area [AOR 1.47 (95% CI 1.002–2.16)]. Furthermore, girls who did not take deworming tablets were likely to be more anemic compared to those who took deworming tablets [AOR 1.33 (95% CI 1.07–1.64)].

## Discussion

The study captured information on vital nutritional indicators such as stunting, thinness, and anemia among unmarried adolescent girls and their predictors in rural Bangladesh. These findings extend beyond national boundaries, reflecting a comparable situation in economically disadvantaged countries across South-East Asia. Our results highlight the positive impact of non-food practices such as using sanitary latrines, practicing hand hygiene, deworming, and television viewing on improved nutritional status and higher Hb levels in adolescent girls. The primary focus of the study was to delve into the underlying causes of undernutrition and anemia among adolescent girls. This aligns with the objectives of the BRAC nutrition program, which seeks to improve conditions of thinness, stunting, and anemia among adolescent girls, as these issues are intricately tied to their reproductive health.

In Bangladesh, a couple of studies had already been conducted to gauge the nutritional status of adolescent girls [48–50]. However, these studies faced limitations such as small sample sizes that might not fully represent all societal segments or the inclusion of only specific indicators of nutritional status. Nonetheless, our current study reveals a troubling finding: the prevalence of stunting and thinness among Bangladeshi adolescent girls has shown minimal improvement over the past decade [48, 50].

The significance of this study is in its exclusive focus on unmarried adolescent girls in the community. These findings strongly support integrating WASH messages with nutrition interventions, aligning with SDG-2 and SDG-6 and also offer substantial evidence to enhance girls’ health and nutrition by aligning with government strategies [30]. In contrast, a study conducted in BRAC health program area included both married and unmarried girls, found associations between anemia and select social factors. These factors might have limited impact on pre-marital undernutrition, anemia, and reproductive health of adolescent girls. Moreover, a study by Chattopadhyay et al. established a link between nutritional status and WASH practices among adolescent girls, albeit lacking data on other essential underlying and causal factors of undernutrition, such as feeding patterns, deworming and morbidity [24], whereas another study found that suffering from diarrhea and dysentery preceding a month of interview were associated with thinness [50]. Notably, a national survey in Bangladesh covered only ages 6–14 years [37].

An important finding, we observed is that stunting predicts thinness, and conversely, thinness predicts stunting in this population. Another study in Bangladesh showed that thinness correlated with low upper arm muscle and fat area Z-scores, rather than stunting [50]. BMI reflects body fat, while height indicates genetic traits influenced by nutrition, environment, and factors like puberty timing [51]. Puberty age affects height gain, influenced by diet, activity, and childhood obesity. Yet, increased growth velocity before puberty can compensate for final height [51]. All these pathways are impacted by energy balance, nutrient quality, especially protein, fat, micronutrients, and healthcare [51]. In Bangladesh, lacking food security hampers preparatory nutrition for pubertal growth, leading to both thinness and stunting. Thus, we observed that both thinness and stunting were associated with each other.

It's important to recognize that factors underlying undernutrition in adolescent girls can differ between countries due to unique contextual elements and study focuses. For instance, in Ethiopia, a study found associations between low BIMZ and factors like age, dietary diversity, and health knowledge, while HAZ was linked to age, household food security, and girls' knowledge, based on community data [52]. In this context, the FFQ quantifies nutrient intake, whereas dietary diversity assesses qualitative aspects of food consumption. In our study, we emphasized the relationship between nutrient intake quantity and girls' nutritional status, recognizing the strong correlation between quantitative and qualitative dietary assessments [53]. Importantly, we did not include qualitative dietary measures. An Ethiopian school-based study identified associations between age and thinness, as well as connections between stunting and factors like menstrual status, food security, and sanitary latrine use [54]. Similarly, other African study tied poor personal hygiene to anemia and underweight, echoing our findings [55]. Another study noted that parental factors' impacted on undernutrition decreased as socioeconomic status improved, which is akin to our study's patterns [56].

Differences in methodology among previous studies have also led to variations in reported anemia prevalence. For example, a study incorporating both married and unmarried girls, using the same Hb measurement method as ours, showed significant differences in anemia prevalence. In contrast, the national micronutrient survey measured iron deficiency anemia using ferritin levels, indicating lower prevalence in children aged 6 to 14 [37]. Notably, the demand for iron notably increases at ages 14 to 15, compounded by iron deficiency from pregnancy and lactation [4]. Despite methodological disparities, these collective findings consistently emphasize the link between early marriage, teenage pregnancy, and their contribution to anemia among adolescent girls.

The current study provides an alarming indication of early marriage and the significant data gap concerning married adolescent girls in rural Bangladesh. Recruiting unmarried girls for interviews was a challenge for current study due to prevalent early marriages. In the rural context of Bangladesh, adolescents have an increased likelihood of being married, experiencing pregnancy, leaving their education, and coming from households with a low socioeconomic status [50]. A nationally representative dataset, covering both married and unmarried girls, was crucial for accurate recruitment planning. However, obtaining current census data was time-consuming, and older data didn't reflect the present community conditions of rural areas [5]. To address this predicament, we chose neighboring villages covered by the BRAC nutrition program to ensure sufficient participants for randomization and interviews.

In urban India, a significant prevalence of anemia and worm infestation was discovered among adolescent girls, emphasizing the need for extra nutritional support, iron-folic acid supplements, and effective worm prevention and management [57]. Notably, we observed protective effects against anemia in those receiving anti-helminthic medication within 6 months. As worms hinder iron absorption, deworming tablets could boost intestinal iron uptake, potentially reducing anemia [58]. Globally, 12% of worm-related disease burden falls on children aged 5 to 14, with anemia risk rising in girls [59]. Our findings stress the urgency of rapid WASH implementation and routine deworming tablet use since childhood. This proactive strategy has the potential to prevent adolescent iron deficiency anemia [60].

Moreover, more extensive adoption of sanitary latrine usage and the practice of washing hands after defecation was noted among adolescent girls. It is noteworthy that, during the survey period, the BRAC WASH program was operational in 61 Districts across Bangladesh. In addition to the effort of the GoB, various other NGOs were also engaged in the study areas to enhance WASH practices. Despite these efforts, a significant proportion of adolescents‒specifically, one-fourth of them displayed gaps in adhering to handwashing with soap after defecation. Additionally, three-fourths of the participants exhibited gaps in handwashing before eating, a practice that could potentially heighten the risk of contracting a range of infectious diseases. Research shows that drinking groundwater is associated with less anemia among women and children even though absence of vitamin C and heme iron in the food [61].We observed that an average 67% of the adolescent girls were drinking ground water (tube well and deep-tube well water). However, from our analysis we have not found any associations between drinking safe water and better Hb level, while average vitamin C intake is 65 and 71 mg/day in the intervention and comparison areas, respectively.

The findings from an observational study indicate that increased nutrient intake, encompassing energy, protein, folate, B12, iron, vitamin C, and red meat, is associated with a reduced likelihood of anemia among women [62]. This study also speculates that fortifying with folic acid could potentially contribute to the lower prevalence of anemia [62]. Notably, in the context of Bangladesh, it is important to highlight that while edible oil is fortified with Vitamin-A, it lacks fortification with iron, folic acid, or other nutrients known to improve anemia [63]. The Vitamins B-6, B-12, A, C, folic acid, and riboflavin are of paramount importance in the synthesis of hemoglobin [64–67]. These nutrients, either directly or indirectly, exert an influence on iron absorption and metabolism. The rich dietary sources of these micronutrients include meat, poultry, fish, milk, legumes, and green leafy vegetables. An analysis of dietary diversity data reveals that roughly three-fourths of adolescent girls included fish, meat, and poultry in their diets during the last 24 h, with one-fourth consuming eggs, one-fourth incorporating green leafy vegetables, and fewer than 30% including dairy products. Moreover, their fruit intake and vitamin C consumption were higher. However, it is vital to recognize that this 24-h dietary pattern does not necessarily reflect their daily eating habits. It is important to emphasize that in the study areas, observations indicated that people typically purchased fish, meat, and poultry only on designated “hat day” (weekly market day) and stored them for the subsequent 2–3 days. On other days of the week, most of them relied on eggs, legumes, dried mung bean pills (known locally as “*daler bori*”), and vegetables, etc., with fish, meat, and poultry being absent from their plates. This pattern persisted until the next “hat day”. Despite this dietary variation, it is conceivable that adolescent girls who consumed more energy might have included more foods that influence hemoglobin production in their diets. This potential association was revealed in our analysis.

However, there are certain limitations inherent to this study. Given that it was a cross-sectional study, it becomes challenging to draw definitive conclusions regarding growth patterns over time. The nature of the study design allows us to generate hypotheses concerning the potential influence of certain variables on the nutritional status of adolescent girls; however, it does not facilitate the exploration of their causal relationships [12].

We converted cooked foods to raw using established methods for nutrient intake calculation [39]. However, our 7-day recall data from a semi-quantitative food frequency questionnaire might have recall bias due to the complexity of Bangladeshi cuisine and regional variations in cooking methods [37]. Although we employed a validated FFQ questionnaire and ensured rigorous training and quality control, the intricacies of Bangladeshi cuisine could lead to underestimation or overestimation of certain foods. Our robust approach is supported by a larger sample size, reflected in normal distribution and statistical measures (SD, SE, and 95% CI) for nutrient intake. Notably, we lack data on nutrient intake for unmarried adolescent girls aged 10–19 years across Bangladesh, limiting our ability to compare their average intake with the national micronutrient survey that evaluated different age groups [37].

We employed a validated method for measuring Hb levels, supported by good sensitivity and specificity [68]. In resource-limited settings, using the hemocue-Hb photometer to assess Hb in capillary or venous blood is widely accepted due to its portability, user-friendliness, and real-time results [69]. Research shows HemoCue's comparability to other methods like Sysmex, KX21N, and Cyanmethemoglobin [68]. Some blood subsamples measured additional indicators (ferritin, serum transferrin receptor, etc.) to enhance results’ validity for iron deficiency anemia. The complementary tests validate hemoglobin (Hb) levels, which are correlated with iron deficiency. Factors such as overhydration, hemoglobinopathies, and chronic health conditions can contribute to a decrease in Hb levels [56, 57]. While this decline may account for anemia, it may not specifically indicate iron deficiency anemia [70]. Despite these considerations, our meticulous process for Hb determination, led by trained field enumerators and specialist teams, aimed to ensure precision and reliability.

Another drawback of our study is the absence of measurements for the physical activity of adolescent girls, a key predictor of the BIMZ score. In urban areas of Bangladesh, overweight parents, limited exercise, and high sedentary lifestyles contribute to obesity among school children [71]. Conversely, rural areas demonstrate a different dynamic, where reduced physical activity in girls directly relates to overweight and obesity [72]. Intriguingly, Saha et al. found that factors like food practices, screen time, gaming, and sleep duration were not significant predictors of obesity of adolescent girls in rural areas [72]. While recognizing this limitation, it's important to emphasize that the lack of physical activity data doesn't diminish the significance of our other findings. They provide valuable insights into various factors influencing the nutritional status of adolescent girls. To comprehensively understand the complexities of teenage health, future research should incorporate assessments of physical activity.

## Implication and conclusions

This study emphasizes the importance of implementing strategies to address undernutrition and anemia before adolescence [11, 60]. Regular deworming tablet administration, coupled with diligent compliance monitoring, could contribute to improving Hb levels in adolescent girls. The Ministry of Health and Family Welfare, under the GoB, has initiated a mass drug administration (MDA) program targeting children aged 5–14 years in schools, excluding those aged 15–19 years [73]. However, recognizing higher dropout rates among adolescent girls [29], it is imperative to extend this initiative to cover all adolescents and involve both governmental and non-governmental partners.

Similarly, an inclusive initiative to enhance knowledge and practices regarding WASH among adolescents, beyond the scope of school sanitation programs, is urgently needed and warrants the involvement of the GoB. In addition to this, the GoB, NGOs, i-NGOs, UN agencies, bilateral and multilateral donors, as well as civil society groups with a significant track record of supporting the health sector, must collaborate to develop and broadcast television programs aimed at raising awareness about these issues. This proactive approach is crucial in breaking the potential cycle of intergenerational undernutrition [60].

Finally, stakeholders in nutrition programs should consider integrating WASH and deworming initiatives and utilizing electronic media to maximize the impact of their investments by addressing issues such as stunting, thinness, and anemia among adolescent girls.

## Data Availability

All relevant data are contained within the manuscript and its accompanying Supporting Information files.
