# Correction to: Water, sanitation and hygiene (WASH) practices and deworming improve nutritional status and anemia of unmarried adolescent girls in rural Bangladesh

**DOI:** 10.1186/s41043-024-00579-3

**Published:** 2024-06-27

**Authors:** Saira Parveen Jolly, Tridib Roy Chowdhury, Tanbi Tanaya Sarker, Kaosar Afsana

**Affiliations:** 1 BRAC James P Grant School of Public Health, BRAC University, 6th Floor, Medona Tower, 28 Mohakhali Commercial Area, Bir Uttom A K Khandakar Road, Dhaka, 1213 Bangladesh; 2 BRAC Research and Evaluation Division, BRAC, 75 Mohakhali, Dhaka, 1212 Bangladesh

**Correction to: J Health Popul Nutr 42, 127 (2023)**

10.1186/s41043-023-00453-8

Following publication of the original article [1], the authors identified errors in Tables 2 and 3. The symbol  ±  appeared twice in Table 2 between mean and (95%.) where it shouldn’t have been indicated. The sub-header Mean  ±  SD was missing from the Table 3 sub-header.

The incorrect Table 2:

**Table 2:** Dietary diversity and nutrients intake and of the adolescent girls by study areas

Variables	**Study area**		p-value
	**Intervention** **n = 811**	Comparison n = 809	
**Dietary diversity**			
Average number of food groups intake during last 24 hrs, Mean ± SD**	3.91 ± 1.25	3.97 ± 1.24	0.889
Dietary diversity score during last 24 hrs, n (%) *			
1 ‒3 food groups (low)	37.85(307)	38.44(311)	0.431
4 ‒6 food groups (average)	59.93(486)	58.54(472)	
7 ‒10 food groups (high)	2.22(18)	3.21(26)	
**Had vitamin and iron rich foods during last 24 hours**			
Vitamin A rich dark green leafy vegetable, n (%) *	20.5 (166)	30.3 (245)	0.000
Organ meat, n (%) *	1.5 (12)	1.2 (10)	0.672
Fish, meat, poultry, n (%) *	73.7 (598)	74.8 (605)	0.630
**Average nutrient intake during last one week**			
Energy, in kcal/day, Mean(95% CI)**	1344.29(1357.64-1418.94)	1403.61(1375.78-1431.43)	0.468
Protein, in g/day, Mean(95% CI)**	45.61(44.37–46.85)	46.36(45.26–47.46)	0.375
Fat, in g/day, Mean (95% CI) **	14.28(13.78–14.78)	14.28(13.77–14.29)	0.998
Carbohydrate, in g/day, Mean (95% CI) **	260.37(254.64-266.11)	264.34(258.83-269.84)	0.328
Calcium, in mg/day, Mean (95% CI) **	635.38(550.01-720.75)	745.07(667.48–822.30)	0.062
Iron, in g/day, Mean (95% CI) **	7.78(7.5–8.07)	8.65(8.22–9.07)	0.001
Zinc, in mg/day, Mean (95% CI) **	8.85(8.31–8.78)	8.68(8.44–9.07)	0.445
Vitamin A, in µg/day, Mean± (95% CI) **	167.95(151.88-184.02)	233.01(207.14-258.19)	0.000
Thiamin, in mg/day, Mean (95% CI) **	1.19(1.16–1.22)	1.18(1.15–1.21)	0.801
Riboflavin, in mg/day, Mean (95% CI) **	0.64(0.61–0.67)	0.67(0.62–0.71)	0.365
Vitamin C, in mg/day, Mean± (95% CI) **	65.00(61.06–68.93)	71.09(67.04–75.14)	0.034
**Intake iron supplementation**			
Intake of iron supplement during last one month, %(n)*	5.2 (42)	4.4(36)	0.493
Frequency of taking iron supplement, %(n)*			
Daily	23.8(10)	38.9(14)	0.322
7 days	33.3(14)	30.6(11)	
< 7 days	42.9(18)	30.6(11)	

*Chi-square test

**Student *t-*test

Hrs = Hours

The correct Table 2:

**Table 2:** Dietary diversity and nutrients intake and of the adolescent girls by study areas

Variables	**Study area**		p-value
	**Intervention** **n = 811**	Comparison n = 809	
**Dietary diversity**			
Average number of food groups intake during last 24 hrs, Mean ± SD**	3.91 ± 1.25	3.97 ± 1.24	0.889
Dietary diversity score during last 24 hrs, n (%) *			
1 ‒3 food groups (low)	37.85(307)	38.44(311)	0.431
4 ‒6 food groups (average)	59.93(486)	58.54(472)	
7 ‒10 food groups (high)	2.22(18)	3.21(26)	
**Had vitamin and iron rich foods during last 24 hours**			
Vitamin A rich dark green leafy vegetable, n (%) *	20.5 (166)	30.3 (245)	0.000
Organ meat, n (%) *	1.5 (12)	1.2 (10)	0.672
Fish, meat, poultry, n (%) *	73.7 (598)	74.8 (605)	0.630
**Average nutrient intake during last one week**			
Energy, in kcal/day, Mean(95% CI)**	1344.29(1357.64-1418.94)	1403.61(1375.78-1431.43)	0.468
Protein, in g/day, Mean(95% CI)**	45.61(44.37–46.85)	46.36(45.26–47.46)	0.375
Fat, in g/day, Mean (95% CI) **	14.28(13.78–14.78)	14.28(13.77–14.29)	0.998
Carbohydrate, in g/day, Mean (95% CI) **	260.37(254.64-266.11)	264.34(258.83-269.84)	0.328
Calcium, in mg/day, Mean (95% CI) **	635.38(550.01-720.75)	745.07(667.48–822.30)	0.062
Iron, in g/day, Mean (95% CI) **	7.78(7.5–8.07)	8.65(8.22–9.07)	0.001
Zinc, in mg/day, Mean (95% CI) **	8.85(8.31–8.78)	8.68(8.44–9.07)	0.445
Vitamin A, in µg/day, Mean (95% CI) **	167.95(151.88-184.02)	233.01(207.14-258.19)	0.000
Thiamin, in mg/day, Mean (95% CI) **	1.19(1.16–1.22)	1.18(1.15–1.21)	0.801
Riboflavin, in mg/day, Mean (95% CI) **	0.64(0.61–0.67)	0.67(0.62–0.71)	0.365
Vitamin C, in mg/day, Mean (95% CI) **	65.00(61.06–68.93)	71.09(67.04–75.14)	0.034
**Intake iron supplementation**			
Intake of iron supplement during last one month, %(n)*	5.2 (42)	4.4(36)	0.493
Frequency of taking iron supplement, %(n)*			
Daily	23.8(10)	38.9(14)	0.322
7 days	33.3(14)	30.6(11)	
< 7 days	42.9(18)	30.6(11)	

*Chi-square test

**Student *t-*test

Hrs = Hours

The incorrect Table 3:

**Table 3:** Nutritional status of the adolescent girls by study area

Variables	Study area		*p*-value
	Intervention *n* = 811	Comparison *n* = 809	
Weight in kg	38.36 ± 8.29	38.50 ± 8.85	0.742
Height in cm	146.57 ± 8.26	146.56 ± 8.46	0.985
^a^ MAC in mm	213.56 ± 28.49	213.42 ± 29.72	0.923
^b^ BMI in kg/m^2^	17.70 ± 2.76	17.74 ± 2.98	0.793
^c^ HAZ- score	­-1.27 ± 1.07	1.24 ± 1.05	0.516
^d^ BMIZ	­0.71 ± 1.07	­0.72 ± 1.11	0.982
Hb in g/dl	12.4 ± 1.3	12.3 ± 1.3	0.529

**Student *t* test

^a^Mid arm circumferences

^b^ BMI = Body Mass Index

^c^Height-for-age Z score

^d^BMI-for-age Z score

The correct Table 3:

**Table 3:** Average nutritional status of adolescent girls by study area

Variables	Study area		*p*-value
	Intervention *n* = 811	Comparison *n* = 809	
	Mean ± SD	Mean ± SD	
Weight in kg	38.36 ± 8.29	38.50 ± 8.85	0.742
Height in cm	146.57 ± 8.26	146.56 ± 8.46	0.985
^a^ MAC in mm	213.56 ± 28.49	213.42 ± 29.72	0.923
^b^ BMI in kg/m^2^	17.70 ± 2.76	17.74 ± 2.98	0.793
^c^ HAZ- score	-1.27 ± 1.07	1.24 ± 1.05	0.516
^d^ BMIZ	0.71 ± 1.07	0.72 ± 1.11	0.982
Hb in g/dl	12.4 ± 1.3	12.3 ± 1.3	0.529

**Student *t* test

^a^Mid arm circumferences

^b^ BMI = Body Mass Index

^c^Height-for-age Z score

^d^BMI-for-age Z score

The correct Tables 2 and 3 have been indicated in this correction article and the original article [1] has been corrected.

## References

[1] Jolly SP, Roy Chowdhury T, Sarker TT (2023). Water, sanitation and hygiene (WASH) practices and deworming improve nutritional status and anemia of unmarried adolescent girls in rural Bangladesh. J Health Popul Nutr.

